# Long‐term outcomes of sentinel node identification using indocyanine green in patients with lung cancer

**DOI:** 10.1111/1759-7714.13737

**Published:** 2020-11-21

**Authors:** Yukikiyo Kawakami, Kazuya Kondo, Naoya Kawakita, Hisashi Matsuoka, Hiroaki Toba, Hiromitsu Takizawa, Mitsuteru Yoshida, Akira Tangoku

**Affiliations:** ^1^ Department of Thoracic, Endocrine Surgery and Oncology Graduate School of Biomedical Sciences, Tokushima University Tokushima Japan; ^2^ Department of Oncological Medical Services Graduate School of Biomedical Sciences, Tokushima University Tokushima Japan; ^3^ Department of Thoracic Surgery Japanese Red Cross Kochi Hospital Kochi Japan

**Keywords:** Indocyanine green, lung cancer, sentinel node

## Abstract

**Background:**

Sentinel node (SN) biopsy is used in the management of numerous cancers to avoid unnecessary lymphadenectomy. This was a clinical exploration/feasibility study of a novel identification technique for SN biopsy using indocyanine green (ICG) fluorescence imaging during lung cancer surgery.

**Methods:**

SN biopsy using ICG was performed on 22 patients who had cT1 or T2N0M0 lung cancer. ICG was injected just around the primary tumor. The fluorescence imaging system enabled visualization of the lymphatic vessels draining from the primary tumor toward the lymph nodes. Fluorescently labeled nodes were dissected, and patients were followed‐up for prognosis and recurrence to confirm the pattern of lymph node metastasis after surgery.

**Results:**

SNs were successfully identified in 16 (72.7%) of 22 patients. A total of 13 of 16 patients had pathological N0 and three had SN metastasis. The median follow‐up time was 92.7 months. Only one patient had no SN metastasis at the postoperative pathological examination and lymph node metastasis during the follow‐up period. The accuracy rate was 93.8% (15/16) and the false‐negative rate was 7.7% (1/13).

**Conclusions:**

SNs were identified by ICG fluorescence imaging, and this technique during lung cancer surgery had good identification and accuracy rates throughout the follow‐up period.

**Key points:**

**Significant findings of the study:**

We attempted to identify sentinel lymph nodes by indocyanine green in lung cancer surgery. The identification rate was 72.7%. The accuracy rate was 100% immediately after surgery, and 93.8% after follow‐up.

**What this study adds:**

Sentinel node biopsy by indocyanine green may be useful for lymph node dissection during lung cancer surgery to avoid unnecessary lymphadenectomy.

## Graphical abstract

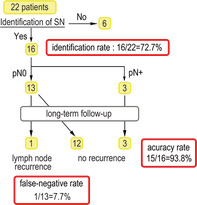

We attempted to identify sentinel lymph nodes by indocyanine green in lung cancer surgery. The identification rate was 72.7%, and the accuracy rate was 100% immediately after surgery. In addition, we followed‐up the cases for a long time to check for lymph node metastasis and verify whether the results of intraoperative sentinel biopsy were correct. After follow‐up had been completed, the false‐negative rate was 7.7% (1/13) and the accuracy rate was 93.8% (15/16) in this study.

## Introduction

Due to the widespread use of thin‐slice computed tomography (CT), small‐sized and/or ground‐glass peripheral lung cancers are being increasingly found. In such cases, radical resection can be performed by segmentectomy instead of lobectomy. Indications for segmentectomy require there to be no regional lymph node metastases. Thoracic surgeons have to confirm that there are no lymph node metastases by making an intraoperative rapid pathological diagnosis during the operation. However, it is difficult to determine which lymph nodes to sample for intraoperative rapid pathological diagnosis. Sentinel node (SN) biopsy has been used in the management of numerous cancers to determine the lymph vessel flow and avoid unnecessary lymphadenectomy.

The concept of the SN is an old but specific technique first reported by Morton *et al*.^1^ They identified lymph vessels and SNs for melanoma using dye. Krag *et al*.^2^ reported that they used radioisotopes (RI) to identify SNs in breast cancer. SN biopsy techniques have been established for melanoma and breast cancer because they were performed first, but they have yet to be established for lung cancer. Little *et al*.^3^ reported that the identification rate of SN in NSCLC was only 47% by the dye method. Several previous studies reported that it was 6.3% to 50.0%, which is poor.^4^, ^5^ Liptay *et al*.^6^ reported the identification rate of SN in NSCLC to be 82% by the RI method. In other studies, the identification rate of SN biopsy by RI was 62.9%–100%.^7^, ^8^, ^9^, ^10^, ^11^, ^12^ Although a phase II trial of the RI method was carried out by multiple institutions, the identification rate of SN was 61.5% and the accuracy rate was 51.2%, which were lower than expected.^13^

In recent years, indocyanine green (ICG) fluorescence has become widely applied in surgery. ICG is a popular diagnostic reagent clinically approved for the examination of hepatic and circulatory function. ICG is less toxic, has no radiation exposure, is cost‐effective, is excreted quickly, and rarely causes allergic reactions. Several studies have previously investigated SN identification using an ICG fluorescence method in lung cancer;^14–17^ however, they had a short follow‐up period after surgery. In this study, we tried to identify the SN by the ICG fluorescence method and followed‐up all patients for a long time after surgery to confirm the precise pattern of lymph node metastasis.

## Methods

### Patients

The protocol was approved by the Human Ethics Review Committee of Tokushima University Hospital on 21 February 2005 (No. 306) and registered in the Japanese clinical trial registry (UMIN000026155). All patients provided their written informed consent. Eligible patients had clinical T1 or T2 N0M0 NSCLC. Other inclusion criteria were Eastern Cooperative Oncology Group performance status of 0 to 2, absence of previous chemotherapy or radiotherapy, and satisfactory hematological, hepatic, and renal function.

All patients were enrolled and treated at our hospital between June 2007 and March 2009. They underwent chest X‐ray, bronchoscopy, chest and abdominal CT, brain magnetic resonance imaging, and/or positron emission tomography‐CT preoperatively. SN biopsy using ICG was performed on 22 patients. The characteristics of the patients are shown in Table 1. The mean age was 67.2 years old. A total of 15 were male (68.2%), 12 were smokers (54.5%) and the mean Brinkman index was 691. The tumor size was 27.1 ± 9.7 mm (mean ± standard deviation). All patients had lobectomy or segmentectomy with lymphadenectomy. Our criteria for the operation procedure were as follows: we performed segmentectomy when the tumor was located in the lower lobe or left upper lobe and was less than 2 cm in diameter, and there was no evidence of SN metastasis. Lobectomy was performed in all other cases.

**Table 1:** Patient characteristics

Age (mean)	67.2 (53–80)
Gender	
Male	15 (68.2%)
Female	7 (31.8%)
Non‐smoker	10 (45.5%)
Smoker	12 (54.5%)
Brinkman index (mean)	691
Histology	
Adenocarcinoma	16 (72.7%)
Squamous cell carcinoma	5 (22.7%)
Large cell carcinoma	1 (4.5%)
Tumor location	
RUL	7
ML	1
RLL	7
LUL	3
LLL	4
Tumor size (mean)	27.1 mm
Operation	
Segmentectomy	7 (31.8%)
Lobectomy	15 (68.2%)

LLL, left lower lobe; LUL, left upper lobe; ML, middle lobe; RUL, right upper lobe; RLL, right lower lobe.

### ICG fluorescence imaging system

Fluorescence images were obtained using the ICG fluorescence imaging system (Olympus, Tokyo, Japan). The light source was a xenon lamp equipped with an excitation filter that permeated light at a wavelength of 690–790 nm, and the detector was a charge‐coupled device (CCD) camera equipped to filter out light with a wavelength below 800 nm. The xenon lamp and excitation filter were aligned on the light source equipment, and the CCD camera was placed at the head for thoracoscopy. The fluorescence signals were sent to a digital video processor. The scope had a diameter of 10 mm. This system can be used in video‐assisted thoracoscopic surgery (VATS), and can switch between normal white light and infrared light for ICG fluorescence.

### SN identification using ICG

**Figure 1:**
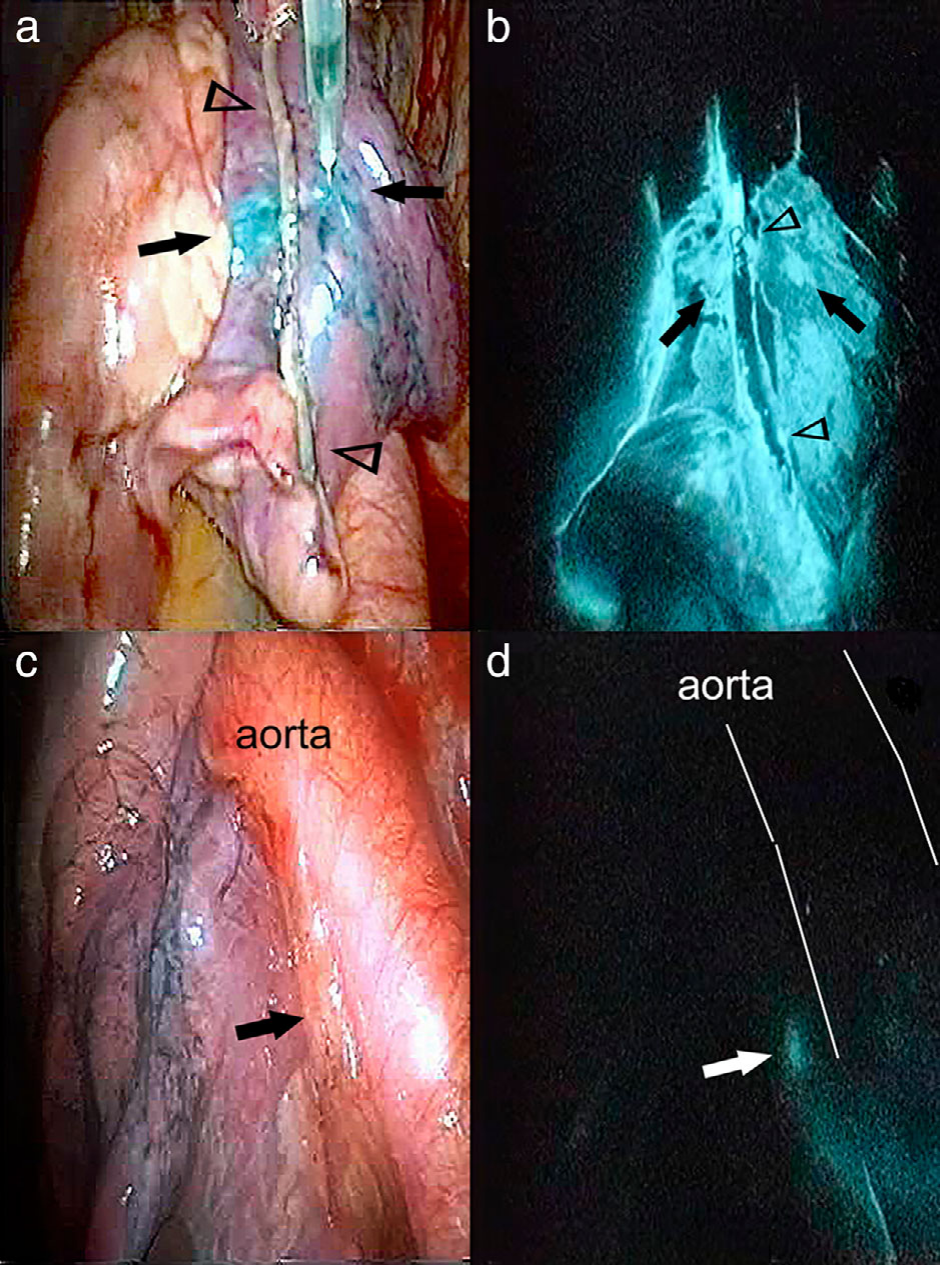
(**a**) The tumor was resected using VATS to confirm lung cancer, as there was no definitive diagnosis preoperatively. During the operative rapid pathological diagnosis, indocyanine green (ICG) (0.5 mL) was injected at two points (arrow) to the proximal part of the stapler line (arrowhead). (**b**) The ICG fluorescence imaging system clearly visualized the ICG‐injected sites (arrow) around the tumor and the spread of the ICG as bright fluorescence. (**c**) The hilar lymph node #10; and (**d**) the lymph node were visualized by fluorescence imaging.

ICG (Diagnogreen; Daiichi Pharm. Co., Tokyo, Japan) at a volume of 0.5–1 mL (5 mg/mL) was injected at 1–4 sites around the primary tumor (Fig 1a). As a result, the ICG dose was 2.5–10 mg. ICG was injected into the collapsed lung for thoracoscopic surgery. The injection site was massaged to spread the ICG for approximately five minutes. The ICG fluorescence imaging system clearly visualized the ICG‐injected sites around the primary tumor and the spread of the ICG as bright fluorescence (Fig 1b). The lymphatic vessels draining the primary tumor toward the lymph nodes were visualized by gradually moving injected dye. The lymph nodes exhibited bright fluorescence. Lymph nodes in the thoracic cavity are often anthracotic under the influence of tobacco, dust and others. Therefore, lymph nodes themselves may be hard to bright on ICG fluorescent imaging. We recognized SN if the tissue around the lymph node was fluorescent, even if the lymph node itself was not fluorescent. First, we detected the lymph flow using the bright fluorescence images. Before lobe resection, we performed systemic dissection of lymph nodes which showed bright fluorescence. After lobectomy, mediastinal lymph node dissection was performed regardless of whether SNs were identified.

### Pathological examination

After SN dissection, SNs were examined by standard histology with hematoxylin‐eosin using conventional methods. They were also examined by conventional intraoperative rapid pathological diagnosis using frozen tissue sections during the operation.

### Statistical analysis

To examine the association between SN identification and patient characteristics (age, gender, Brinkman index, tumor location [right vs. left, upper and middle lobes vs. lower lobe], tumor size, pathology [adenocarcinoma vs. squamous cell carcinoma] and operation [lobectomy vs. segmentectomy]), we used the unpaired *t*‐test and chi‐square test.

## Results

### SN identification rate

SNs were successfully identified in 16 of 22 patients (identification rate: 72.7%) (Table 2). The lymphatic flow was clearly observed in all 16 patients. Among the six patients in whom the SNs were not identified, some did not show lymphatic flow and the SN was not identified, even though the injected ICG was visible in others. In 13 of 16 patients, there was no evidence of metastasis in SNs or non‐SNs. There was SN metastasis detected in three patients (Cases 6, 12 and 14).

**Table 2:** Case details

No	B.I.	His.	Location	Tumor size	Operation	SN	Dissected mediastinal lymph node stations	pN	Follow‐up period (months)	Recurrence (months after surgery)	Prognosis	Pathological findings of LN
1	0	ad	lt.S^10^	31 mm	lob	#10	#4L, 5, 6, 7, 8, 9	0	62.9	None	Alive	N0 (0/20)
2	4000	sq	rt.S^9 + 10^	48 mm	lob	N.D.	#4R,7, 8, 9	0	107.9	None	Alive	N0 (0/32)
3	800	ad	rt.S^2^	28 mm	lob	#11s	#4R, 7	0	61.3	None	Alive	N0 (0/13)
4	0	ad	rt.S^8^	15 mm	seg	N.D.	#7, 8	0	74.1	None	Alive	N0 (0/6)
5	0	ad	rt.S^2^	22 mm	lob	#4R	#4R	0	54.9	lymphnode (21.5)	Dead	N0 (0/26)
**6**	**1400**	**ad**	**lt.S** ^**3**^	**20 mm**	**seg**	**#12**	**#5**	**1**	**148.0**	**None**	**Alive**	**#5(0/6), #12(1/1), #13(3/10)**
7	740	ad	lt.S^1 + 2^	31 mm	lob	#10	#5, 6, 7	0	103.2	Lung (37.9)	Dead	N0 (0/18)
8	1280	ad	lt.S^9^	19 mm	seg	#12	#8, 9	0	94.2	None	Alive	N0 (0/15)
9	0	ad	rt.S^8^	17 mm	seg	#7	#7	0	146.0	Lung (33.3)	Alive	N0 (0/15)
10	1620	sq	lt.S^8^	47 mm	lob	N.D.	#7, 8, 9	0	17.4	Bone (5.2)	Dead	N0 (0/9)
11	660	ad	rt.S^6^	15 mm	seg	#7	#7, 8, 9	0	17.5	Adrenal gland (17.0)	Dead	N0 (0/19)
**12**	**0**	**ad**	**rt.S** ^**4**^	**33 mm**	**lob**	**#11s**	**#4R, 7**	**1**	**144.7**	**Pleura (73.2)**	**Alive**	**#4R(0/15), #7(0/10),11s(1/6), #12(0/1), #13(0/2)**
13	680	ad	lt.S^1 + 2^	18 mm	seg	N.D.	#5, 6	0	120.2	None	Alive	N0 (0/10)
**14**	**720**	**ad**	**lt.S** ^**10**^	**16 mm**	**lob**	**#10**	**#5, 7**	**2**	**134.4**	**None**	**Alive**	**#5(0/10), #7(1/2), #10(1/1), #11(1/14), #12(0/10), #13(0/1)**
15	0	sq	rt.S^3^	37 mm	lob	#4R	#4R, 7	0	50.9	Adrenal gland(6.3)	Dead (other disease)	N0 (0/38)
16	0	ad	rt.S^2^	17 mm	lob	#12	#4R, 7	0	85.0	None	Alive	N0 (0/17)
17	0	ad	rt.S^2^	26 mm	lob	#4R	#4R	0	59.7	None	Alive	N0 (0/10)
18	0	ad	rt.S^10^	25 mm	lob	N.D.	#4R, 7	0	102.5	None	Alive	N0 (0/19)
19	0	ad	rt.S^6^	28 mm	seg	#10	#7	0	132.5	None	Alive	N0 (0/20)
20	600	sq	rt.S^2^	40 mm	lob	N.D.	#4R, 7	0	111.8	None	Alive	N0 (0/11)
21	1500	sq	rt.S^8^	30 mm	lob	#10	#4R, 7	0	129.0	None	Alive	N0 (0/22)
22	1200	la	rt.S^1^	34 mm	lob	#11s	#4R, 7	0	81.1	None	Alive	N0 (0/12)

B.I., Brinkmann Index; His., histology; SN, sentinel node; lob, lobectomy; seg, segmentectomy; N.D., not detected; ad, adenocarcinoma; sq., squamous cell carcinoma; la, large cell carcinoma.

### Representative patients with SN metastasis

**Figure 2:**
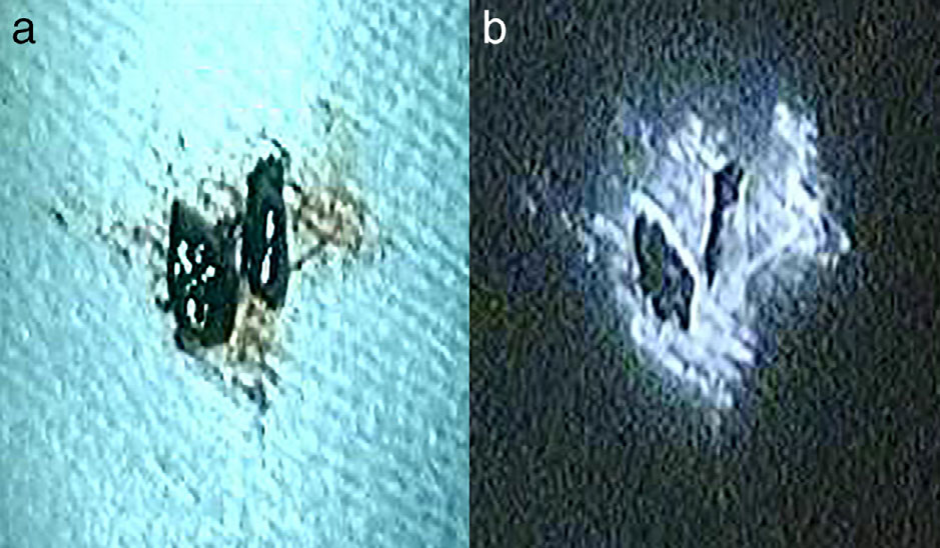
(**a**) The dissected lymph node #10 (SN). (**b**) The lymph vessels and adipose tissue around the lymph nodes emitted ICG fluorescence ex vivo. The interior portion of the lymph nodes did not emit bright fluorescence, but the external portion or the circuit of the lymph nodes did.

In Case 14, the primary tumor was 16 mm in diameter and located in the left S^10^. It was resected using VATS and the operative rapid pathological diagnosis demonstrated adenocarcinoma. Immediately after intraoperative ICG injection, the fluorescence imaging system enabled visualization of the hilar lymph node (#10) (Fig 1c,d). The ICG flowed from the primary tumor via #10 to the subcarinal lymph node (#7). We defined #10 as the SN. The intraoperative rapid pathological diagnosis revealed metastasis in #10 and #7. We decided to convert from segmentectomy to lobectomy, and performed aortic and subcarinal lymph node dissection. Figure 2 shows the resected lymph node #10 ex vivo. The surrounding tissue often fluoresce strongly, not the lymph nodes themselves. There was metastasis in SN (#10), the inferior interlobar lymph node (#11i), and #7 LN. The subaortic lymph node (#5), lower lobar lymph node (#12), and segmental lymph node (#13) had no evidence of metastasis. The patient is alive 134 months after the operation and has had no recurrence.

In Case 6, the primary tumor was 20 mm in diameter and located in the left S^3^. The ICG flowed from the primary tumor via the upper lobar lymph node (#12) to the subaortic lymph node (#5). We defined #12 as the SN. The operative rapid pathological diagnosis revealed metastasis in #12 LN. However, lobectomy was not possible due to poor respiratory function, and segmentectomy was performed and only #5 that fluoresced by ICG was dissected. Following pathological examination, there was metastasis detected in #12u and the segmental lymph node (#13), whereas no evidence of metastasis in #5. The patient is alive at 148 months after the operation and has had no recurrence.

In Case 12, the primary tumor was 33 mm in diameter and located in the right S^4^. The ICG flowed from the primary tumor via the superior interlobar lymph node (#11s) to the right lower paratracheal lymph node (#4R). We defined #11s as the SN. We performed right middle lobectomy with lymph node dissection. There was only metastasis detected in the SN (#11s), and #4R and #7 had no metastasis. Disseminated lesions recurred 73 months after the operation, but the patient is alive at 144 months due to epidermal growth factor receptor‐tyrosine kinase inhibitor (EGFR‐TKI) treatment. Before follow‐up, the false‐positive rate was 0% (0/16) and the accuracy rate was 100% (16/16) in this study.

### Follow‐up of prognosis and recurrence

We followed‐up all patients with regard to prognosis and recurrence to confirm the pattern of lymph node metastasis after surgery. The median follow‐up time was 92.7 months (17.4–148.0 months). Seven (31.8%) of 22 patients had recurrence. Hematogenous metastasis was noted in five (lung metastasis; two, adrenal metastasis; two, bone metastasis; one), dissemination was noted in one, and lymph node metastasis was noted in one. Four of seven patients with recurrence died from lung cancer.

Of the 16 patients in whom the SN was identified, 15, including three with metastasis to the SN, had no postoperative lymph node recurrence. Only one patient (Case 5) had lymph node metastasis during follow‐up. In this case, the primary tumor was located in the right S^2^. We defined #4R as the SN and performed lobectomy and superior mediastinal lymph node dissection. The postoperative pathological examination confirmed no metastasis in all resected lymph nodes. Recurrence in the right upper paratracheal lymph node (#2R) was found at one year and nine months after surgery. Four months later, it had spread to the left supraclavicular lymph node. In 15 (93.8%) of 16 patients with SNs, their metastatic patterns predicted the metastatic pattern of all lymph nodes. After follow‐up was completed, the false‐negative rate was 7.7% (1/13) and the accuracy rate was 93.8% (15/16) in this study.

### Identification of the SN and clinical findings

We examined the association between SN identification and patient characteristics (age, gender, Brinkman index, tumor location [right vs. left, upper and middle lobe vs. lower lobe], tumor size, pathology [adenocarcinoma vs. squamous cell carcinoma] and operation [lobectomy vs. segmentectomy]). There was no significant association between SN identification and patient characteristics.

## Discussion

SN biopsy has been used in the management of numerous cancers to avoid unnecessary lymphadenectomy. In recent years, there have been reports of lobe‐specific selective lymph node dissection in lung cancer. The results in some studies have been reported to be similar to those of systematic lymph node dissection,^18^, ^19^ but others reported more lymph node recurrence and a poorer prognosis than systematic dissection.^20^, ^21^ Thus, as there is no consensus, SN identification for lymph vessel flow may be necessary to reduce the extent of lymph node dissection in each case.

We tried to identify the SN using an ICG fluorescence method. The results of SN identification by the ICG fluorescence method in NSCLC in four previous studies are shown in Table 3.^14^, ^15^, ^16^, ^17^ The SN identification rate in our study was 72.7%, similar to that reported from other institutions (80.3%–83.7%). Although the results by Gilmore *et al*.^16^ were low (45.5%), they conducted an optimized ICG dose escalation trial for SN identification and reported that SN identification increased according to the ICG dose, with a success rate of 89% at 1000 mg or greater. The use of a sufficient amount of ICG can detect SNs in 70%–80%. The metastatic rate of SNs in this study was 3/15 (19%). A wide‐ranging metastatic rate of SNs (4%–40%) has been previously reported. As the study by Gilmore *et al*. included large tumors (4, 6 and 6.5 cm), the metastatic rate of SNs may have been higher (40%). On the other hand, other studies included stage I cancer and the metastatic rate was relatively low (4.0%–9.7%).

**Table 3:** Results of SN identification using ICG

						Identification		Metastasis		False‐negative		Accuracy		
Author	Institution	Research period	Publish	Cases	ICG amount (mg)	Cases	%		%		%		%	Pathological examination
Yamashita *et al*. (2011)^14^	Oita University	January 2009–September 2009	Nov 2009	31	10	25	80.6	1	4.0	0/24	0	25/25	100	Standard histological examination
Yamashita *et al*. (2012)^15^	Oita University	January 2009–December 2010	Nov 2011	61	10	49	80.3	2	4.1	1/49	2.1	48/49	98.0	RT‐PCR of CK‐19 gene
Gilmore *et al*. (2013)^16^	Brigham & Women's Hospital Beth Israel Deaconess Medical Center	February 2009–February 2013	Apr 2013	33	†	15	45.5	6	40.0	0/9	0	15/15	100	IHC AE1/AE3 cytokeratin, cytokeratin 7, thyroid transcription factor 1
Nomori *et al*.(2007)^12^	Kameda Medical Center	January 2013–May 2015	Aug 2015	135	10	113	83.7	11	9.7	3/113	2.3	110/113	97.3	IHC anticytokeratin AE1/AE3 antibodies
Our study	Tokushima University	June 2007–March 2009		22	2.5–10	16	72.7	3	18.8	0/13	0	16/16	100	Standard histological examination
										1/13 ^‡^	7.7 ^‡^	15/16 ^‡^	93.8 ^‡^	

^†^ Dose escalation trial.

^‡^ After follow‐up.

CK‐19, cytokeratin 19; ICG, indocyanine green; IHC, immunohistochemistry; RT‐PCR, reverse transcription‐polymerase chain reaction; SN, sentinel node.

In postoperative diagnosis, the false‐positive rate was 0% and the accuracy rate was 100%, similar to results reported from other institutions. In other studies, the false‐positive rate was 0%–2.3% and the accuracy rate was 97.3%–100%. The “true positive rate” should not change by following‐up the patients, whereas the “true false‐negative rate” and “true accuracy rate” may change during follow‐up. Previous studies had a shorter period of follow‐up after surgery. We followed‐up all patients for 92.7 months (mean) after surgery to confirm the precise pattern of lymph node metastasis. A total of 12 patients had no SN metastasis at the intraoperative rapid or postoperative pathological diagnosis and no lymph node metastasis during the follow‐up period. Only one patient exhibited no SN metastasis at the postoperative pathological diagnosis and lymph node metastasis during the follow‐up period. Recurrence in #2R was noted at one year and nine months after surgery. In our study, the “true false‐negative rate” was 7.7% (1/13) and the “true accuracy rate” was 93.8% (15/16). Three patients showed SN metastasis at the intraoperative rapid or postoperative pathological diagnosis, but had no lymph node metastasis during the follow‐up period.

There was no significant association between SN identification and patient characteristics (age, gender, Brinkman index, tumor location, tumor size, pathology, and operation method) in our study, consistent with the study by Nomori *et al*.^17^

The limitations of the present study were the small number of patients and single‐institution design. Many patients via a multi‐institutional study may lead to more accurate results. We used the conventional pathological method for the intraoperative rapid or postoperative pathological diagnosis. In future, we may be able to precisely detect SN metastasis using reverse transcription‐polymerase chain reaction (RT‐PCR) or immunohistochemical analysis of CK‐19 and CK‐7.

We also tried another SN identification method using a CT contrast agent. Lymphography with transbronchial injection of a water‐soluble extracellular CT contrast agent, iopamidol, was performed by broncoscopy before surgery.^22^ The identification rate of the SN was 92.3% (12/13 patients). The metastatic rate was 16.7% (2/12 patients). The accuracy rate was 100% (12/12 patients) and the false negative rate was 0% (0/10 patients). We therefore consider it useful as a preoperative simulation tool for SN dissection. Compared with CT lymphography, the intraoperative ICG fluorescence method is advantageous in that bronchoscopy is unnecessary.

In conclusion, our identification technique for SN using ICG fluorescence imaging during lung cancer surgery had good identification and accuracy rates throughout the follow‐up period. This technique may be useful for lymph node dissection during lung cancer surgery to perform segmentectomy and avoid unnecessary lymphadenectomy.

## Disclosure

The authors report no conflict of interest.
